# Impact of a High-Power Light-Emitting Diode Unit With Reduced Curing Times on the Shear Bond Strength of Orthodontic Brackets and Their Adhesive Remnant Index Scores

**DOI:** 10.7759/cureus.39855

**Published:** 2023-06-02

**Authors:** Neelapala Rohini, Ghanta Sunil, RSVM Raghu Ram, Inuganti Ranganayakulu, Balanaga Vijaya Krishna

**Affiliations:** 1 Orthodontics and Dentofacial Orthopaedics, GSL Dental College & Hospital, Rajamahendravaram, IND

**Keywords:** ari score, curing time, shear bond strength, led curing unit, high-intensity led

## Abstract

Aim and objectives: This study aims to evaluate the curing time minimally required for bonding stainless-steel (SS) brackets using a high-power light-emitting diode (LED) light curing unit (LCU) and examine the debonded enamel surface for adhesive remnant.

Materials and methods: Based on the LED LCU and curing time employed, 80 human maxillary first premolar teeth were equally segregated into four groups. Three groups were cured using a high-power LED unit (Guilin Woodpecker Medical Instrument Co., Ltd., Guilin, Guangxi, China) for one, two, and three seconds. The fourth group served as a control and was bonded with another intensive LED unit (Elipar™ S10 LED Curing Light; 3M, Saint Paul, Minnesota, United States) for 20 seconds. Transbond™ XT Light Cure Adhesive (3M, United States) adhesive was used for bonding the SS brackets. All the samples were exposed to shear bond strength (SBS) testing after a 24-hour immersion period in distilled water at 37°C. A stereomicroscope was used to examine and score the adhesive remnant on the debonded surface using a modified adhesive remnant index (ARI). Kruskal-Wallis-ANOVA and post-hoc Mann-Whitney U tests for multiple pairwise comparisons were performed to analyze the data.

Results: Time and intensity significantly affected the SBS (P<0.001). A higher SBS value (16.04 megapascals (MPa)) was obtained in the six-second group when compared to the three-second (11.58 MPa), one-second (10.69 MPa), and 20-second control (13 MPa) groups. The ARI was significantly affected by the curing method.

Conclusions: Higher SBSs were recorded for the six-second group using the high-power LED. A greater ARI score is associated with a reduced curing duration and vice versa.

## Introduction

Bracket bonding is a significant and time-consuming element in orthodontic treatments [1]. The integrity of the bonding of orthodontic brackets and the amount of time spent on therapy during bonding are concerns shared by all orthodontists. Reducing the time required for bonding without compromising the bond strength can improve patient comfort and an orthodontist’s treatment efficiency [2]. To achieve that, several light curing systems, such as quartz-tungsten-halogen, argon laser, and plasma arc light curing units (LCUs), have been introduced with a gradual reduction in curing periods. However, these curing systems are large and expensive with a limited lifetime [3]. To overcome these drawbacks, Mills introduced a novel LCU in 1995 that polymerizes light-activated dental materials using solid-state light emitting diode (LED) technology. They have a lifespan of more than 10,000 hours, are resistant to impacts, consume very less power, and run on batteries that are rechargeable with an ergonomic design and lightweight [4]. These conventional LED units are used at a 20-second exposure per bracket, which however requires 10 minutes for bonding.

Accordingly, new-generation, high-power LED LCUs have evolved to further reduce the bonding time without compromising the bond strength. However, a recent study using a new high-power LED (3200 mW/cm^2^), reported a mean shear bond strength (SBS) of 21.56 megapascals (MPa) with a six-second curing time and 15.79 MPa with a three-second curing time, suggesting that clinicians would certainly benefit from this short-duration exposure. However, the LED light used is relatively expensive and also reported enamel loss on debonding due to the high SBS [2]. Recently, an economical, high-power (2400 mW/cm^2^) LED unit has been introduced into the market, promising an adequate SBS at a six-second curing time. However, an SBS, with further reduction in the curing time, such as one and three seconds, has not been tested with economical high-power LED to date. Moreover, adhesive failures have not been evaluated during bracket debonding. As a result, the goal of this study is to examine how the bond strength of metal brackets and the quantity of adhesives left on the tooth surface after debonding are affected by a shorter curing period.

## Materials and methods

Methodology

The study was conducted at the GSL Dental College & Hospital, Rajamahendravaram (Rajahmundry), India, and approved by the Institutional Ethics Committee of GSL Dental College & Hospital (approval number: GSLDC/IEC/2019/017). A power analysis was used to determine that a sample size of 80 would provide a statistical significance (alpha level) of 0.05 at 80% power. Eighty human maxillary first premolar teeth extracted for therapeutic purposes were standardized in accordance with Almeida et al. [2], which includes morphologically and anatomically well-defined teeth with a complete buccal surface. Teeth with enamel fractures, fluorosis, and caries and restored teeth were excluded according to Ulker et al. [5]. All the samples were processed as per the technical specification ISO/TS 29022:2013 (beveled-edge shear bond strength test), followed by storing in distilled water until the time of bonding, which was no more than six months. The tooth on its buccal crown aspect was cleaned, and pumice was used for polishing for 10 seconds, rinsed with water for 10 seconds, and dried for another 10 seconds with oil-free compressed air (three-way syringe). The sample was randomly allocated into four groups (20 each).

To facilitate identification, the roots of the teeth were placed in self-curing colored acrylic blocks (individualized for each group) up to the cementoenamel junction leaving the crowns. The teeth were aligned with their buccal surfaces perpendicular to the block’s base. Of the four groups, Group I, Group II, and Group III were the intervention groups, which were cured using a new high-power LED unit (2400 mW/cm^2^) (Woodpecker iLED, Guilin Woodpecker Medical Instrument Co., Ltd., Guilin, Guangxi, China) for one, three, and six seconds, respectively. Group IV served as control and was cured using a conventional LED unit (1400 mW/cm^2^) (Elipar™ S10 LED Curing Light; 3M, Saint Paul, Minnesota, United States) for about 20 seconds. Etchant (3M ESPE Scotchbond Etchant; 3M, United States) and primer (Transbond™ XT Light Cure Adhesive; 3M, United States) were applied to all the samples in accordance with the manufacturer’s instructions to condition the buccal surface of each tooth. A layer of adhesive (Transbond XT) was applied on the base of maxillary first premolar pre-adjusted edgewise stainless-steel brackets (Unitek™ Gemini Twin Brackets; 3M, United States) and pressed gently on the middle of the buccal surfaces, and an explorer was used to remove the extra adhesive. The adhesive was cured for about one and three seconds, with a single exposure on the mesial side of the bracket using a new high-power LED LCU (2400 mW/cm^2^) (Woodpecker iLED) for Groups I and II. The adhesive was cured for six seconds with a new high-power LED unit (2400 mW/cm^2^) (Woodpecker iLED) and 20 seconds using a conventional LED unit (1400 mW/cm^2^) (Elipar LED) by positioning the curing tip on the bracket/tooth interface at the mesial surface for half of the total curing period and the rest of the curing time on the distal surface for Groups III and IV, respectively.

During light exposure, the curing point was placed as near as feasible to the tooth-bracket interface. Afterward, all the samples were incubated at 37℃ for 24 hours in a closed glass container in distilled water. Using a universal testing machine (UTM) with a 1 kN load cell and a crosshead speed of 0.5 mm/min, an SBS testing was carried out. The sample was locked onto the lower jaw compartment, and a sharp end of the rod was locked on the upper jaw compartment of the UTM machine. The area between the base and the wings of the bracket that is attached to the teeth was incised by the sharp end of the rod when it was stressed in an occluso-gingival manner. For each sample, the force in Newton (N) at which the orthodontic bracket debonded was noted. The SBS for each sample was computed by dividing the force by the bracket pad surface area. Megapascal (MPa) unit was used to measure the force per unit area.

Evaluation of surface characteristics

The debonded enamel surfaces were examined under a light stereomicroscope (Infinity IOX-6745 Stereo Zoom Microscope, I7 Opto Electronics Inc., Ambala, Haryana, India) at a 20× magnification to determine the mode of failure. The viewed image was subsequently saved to a desktop computer, where the modified adhesive remnant index (ARI) scores were evaluated and scored based on the adhesive that remained on the tooth surface after debonding. The modified ARI scores of the four groups were tabulated using the findings of the SBS tests.

Statistical analysis

The obtained data were analyzed using IBM SPSS Statistics for Windows, Version 20 (Released 2011; IBM Corp., Armonk, New York, United States). ANOVA using Kruskal-Wallis and post-hoc Mann-Whitney U tests for multiple pairwise comparisons were performed to analyze the data. P≤0.05 was considered statistically significant.

## Results

Significant differences were statistically observed in the SBS values among the four groups (P<0.001). The highest SBS was shown by the Woodpecker iLED with a six-second curing time, followed by Elipar LED with a 20-second curing time (Table 1).

**Table 1:** Comparison of the SBSs of brackets between the control and experimental groups * Kruskal-Wallis analysis of variance (ANOVA); level of significance P<0.001 LED = light-emitting diode Woodpecker iLED, Guilin Woodpecker Medical Instrument Co., Ltd., Guilin, Guangxi, China Elipar™ S10 LED Curing Light; 3M, Saint Paul, Minnesota, United States

LED curing unit	Curing time	Sample size	Mean ± SD	P value
Woodpecker iLED (Experimental group)	1 sec	20	10.69 ± 2.07	<0.001*
	3 sec	20	11.58 ± 2.04	
	6 sec	20	16.04 ± 2.59	
Elipar LED (Control group)	20 sec	20	13 ± 2.72	

Multiple pairwise comparisons revealed that Woodpecker iLED with a six-second curing time had significantly higher SBS values compared to all the other study groups, including Elipar LED with a 20-second curing time. The SBS reported by Woodpecker iLED with a three-second curing time did not exhibit a significant difference from that of Elipar LED with a 20-second curing time. Moreover, no significant difference was observed between between Group I and Group II (Table 2).

**Table 2:** Multiple pairwise comparisons of the SBSs of brackets between the study groups * Mann-Whitney U test; level of significance P<0.001 SBS = shear bond strength Woodpecker iLED, Guilin Woodpecker Medical Instrument Co., Ltd., Guilin, Guangxi, China Elipar™ S10 LED Curing Light; 3M, Saint Paul, Minnesota, United States

Parameter	Reference group	Comparison group	Mean difference	P value
SBS (MPa)	1 sec (Woodpecker iLED)	3 sec (Woodpecker iLED)	-0.88	0.148
		6 sec (Woodpecker iLED)	-5.35	<0.001*
		20 sec (Elipar LED)	-2.31	<0.001*
	3 sec (Woodpecker iLED)	1 sec (Woodpecker iLED)	0.88	0.148
		6 sec (Woodpecker iLED)	-4.46	<0.001*
		20 sec (Elipar LED)	-1.42	0.083
	6 sec (Woodpecker iLED)	1 sec (Woodpecker iLED)	5.35	<0.001*
		3 sec (Woodpecker iLED)	4.46	<0.001*
		20 sec (Elipar LED)	3.04	<0.001*
	20 sec (Elipar LED)	1 sec (Woodpecker iLED)	2.31	<0.001*
		3 sec (Woodpecker iLED)	1.42	0.083
		6 sec (Woodpecker iLED)	-3.04	<0.001*

Based on the comparison between the study groups, a modified ARI score of 3 was predominant. Groups III and IV had significantly lesser ARI scores than Groups I and II (Table 3).

**Table 3:** Modified ARI score frequency distribution between the research groups Modified ARI: score 0 = no adhesive left on the tooth; score 1 = 1%-25% of adhesive left on the tooth; score 2 = 26%-50% adhesive left on the tooth; score 3 = 51%-75% adhesive left on the tooth; score 4 = 76%-99% adhesive left on the tooth; score 5 = all adhesive left on the tooth with a distinct impression of the bracket mesh LED = light-emitting diode; ARI = adhesive remnant index Woodpecker iLED, Guilin Woodpecker Medical Instrument Co., Ltd., Guilin, Guangxi, China Elipar™ S10 LED Curing Light; 3M, Saint Paul, Minnesota, United States

LED curing unit	Curing time	Modified ARI score					
		0	1	2	3	4	5
Woodpecker iLED (Experimental group)	1 sec	2	1	3	7	3	4
	3 sec	2	2	3	3	3	7
	6 sec	3	3	7	6	1	0
Elipar LED (Control group)	20 sec	1	5	2	6	3	3

## Discussion

One of the most time-consuming orthodontic treatments is bonding brackets. The most crucial component in generating sufficient cohesive composite resin strength is polymerization, which is directly proportional to the entire quantity of light energy that the resin absorbs. The intensity of light multiplied by the exposure time is equal to the total energy of light. A greater total light energy causes the resin to have higher fracture toughness and flexural strength, which is then transferred onto the brackets bonded to the teeth, resulting in a higher SBS [3]. High-power light has the advantage of allowing the composite to receive the same amount of total light energy in a much shorter amount of time, improving the effectiveness of an orthodontist’s treatment and comfort of the patient. Because the degree of the conversion rate for composite adhesive is directly proportional to the power absorbed by its photoactivators, the results of the current investigation shows that the mean SBS increased as the curing period increased. Similar findings have been reported by other researchers [4, 6-8]. Reynolds mentioned that the “clinically acceptable” SBS values for bonding brackets range from 5.9 to 7.8 MPa [9]. However, obtaining an adequate SBS for orthodontic needs without fracturing the enamel during debonding would be beneficial.

In the current investigation, reducing the curing time to one second produced a similar SBS (10.69 MPa) to that with a 10-second curing time in other studies, which might be safer in terms of avoiding enamel fracture [6,7,10]. By contrast, a lower mean SBS of 6.426 ± 1.839 MPa was reported under similar conditions [11]. No other studies were documented using high-power LED LCUs at as low as one-second curing time. However, even the lower-bound SBS value obtained in our investigation after curing for one second was greater than the ideal criterion proposed by Reynolds [9], proving it efficient for orthodontic purposes. Reducing the curing time to three seconds created an utmost comparable SBS (10.69 MPa) to earlier studies using a 10-second curing period [10,12]. However, curing for three seconds with a higher-power (3200 mW/cm^2^) LED produced distinct SBS values (15.79 and 11.43 MPa) [2,13]. Similarly, higher SBSs were reported on curing for six seconds at increased intensities (21.56 MPa at 3200 mW/cm^2^ [2] and 33 MPa at 5000-6000 mW/cm^2^) [14]. On the contrary, lower SBSs were reported on curing for the same time using low-power density LEDs [15,16]. Moreover, the SBS produced at a six-second curing time (16.04 MPa) was similar to that at a 40-second curing time, which might be adequate to resist orthodontic and masticatory forces at a shorter curing duration [10,17].

In the present study, statistically significant highest and least SBS values were reported with Group III and Group I, respectively. The mean bond strength obtained for Group III was considerably higher than that for Group IV. No statistically significant difference between Groups II and IV was observed. Thus, curing with high-power LED can significantly shorten the curing time. Lower SBSs were reported with the high-power LED for one and three seconds, but they did not differ significantly. Nonetheless, all experimental and control groups produced SBSs that can withstand normal orthodontic forces. However, curing for one and three seconds should be done with caution due to reduced time duration. The statistics obtained from the present in vitro study, however, are validated only when related within the same study groups and cannot be generalized to other clinical situations or laboratory values.

In bracket bonding, adhesion must be sturdy to keep the bracket in position until the treatment is completed while also allowing for safe debonding. Extremely high values, on the other hand, may increase the risk of enamel breakage during debonding. Adhesive failure at the composite-bracket interface can be the safest pattern to escape enamel fracture during debonding, and the present study predominantly reported an ARI score of 3, which possibly reduced the enamel trauma [2]. In the current investigation, the ARI scores of the groups with lower SBS values (Groups I and II) were observed to be substantially more (3, 4, and 5) than the scores (0, 1, and 2) of groups with more SBS values (Groups III and IV).

Due to the reduced adhesion to the bracket base, it is likely that insufficient curing with high-power LED for one and three seconds caused a lower SBS and a higher ARI score. These findings are congruent with those of Cerekja and Cakirer [1]. Enamel fracture risk was deemed to be higher for SBS values over 13 MPa. However, the current investigation discovered enamel fractures/cracks with SBS values higher than 19.5 MPa [2]. A total of three samples were observed with enamel fractures subjected to the SBS test. Two of them were light-cured for six seconds using Woodpecker iLED light, with SBS values of 19.53 and 20.45 MPa, and one for 20 seconds using Elipar LED light, with an SBS value of 20.33 MPa. However, caution should be exercised in extrapolating the obtained data to the clinical context because these in vitro results might not accurately reflect the conditions in the oral cavity.

Limitations

As this is an in vitro investigation, the results cannot be directly compared to clinical performance. Variations in the experimental conditions might differ in the SBS values. The debonding forces of brackets from in vivo investigations differ to those from in vitro investigations. The increase in pulpal temperature using high-power LED units was not measured.

Further scope

As the oral conditions influence the bond strength, further clinical studies are necessary for validation. The rate of pulpal damage using high-power LED units needs further studies.

## Conclusions

Significantly higher SBSs were recorded on curing for six seconds with the high-power LED. The SBSs in Groups IV and II did not differ statistically. The mean SBSs recorded in Groups I and II were sufficient to resist orthodontic forces and were the safer values for avoiding enamel fracture. Curing times that are shorter are related to a higher ARI score and vice versa.
